# Supplementary figures and images for: Enhanced antioxidant activity of Chenopodium formosanum Koidz. by lactic acid bacteria: Optimization of fermentation conditions
Source: PLoS One. 2021 May 11;16(5):e0249250. doi: 10.1371/journal.pone.0249250 (PMC8112705; doi:10.1371/journal.pone.0249250)

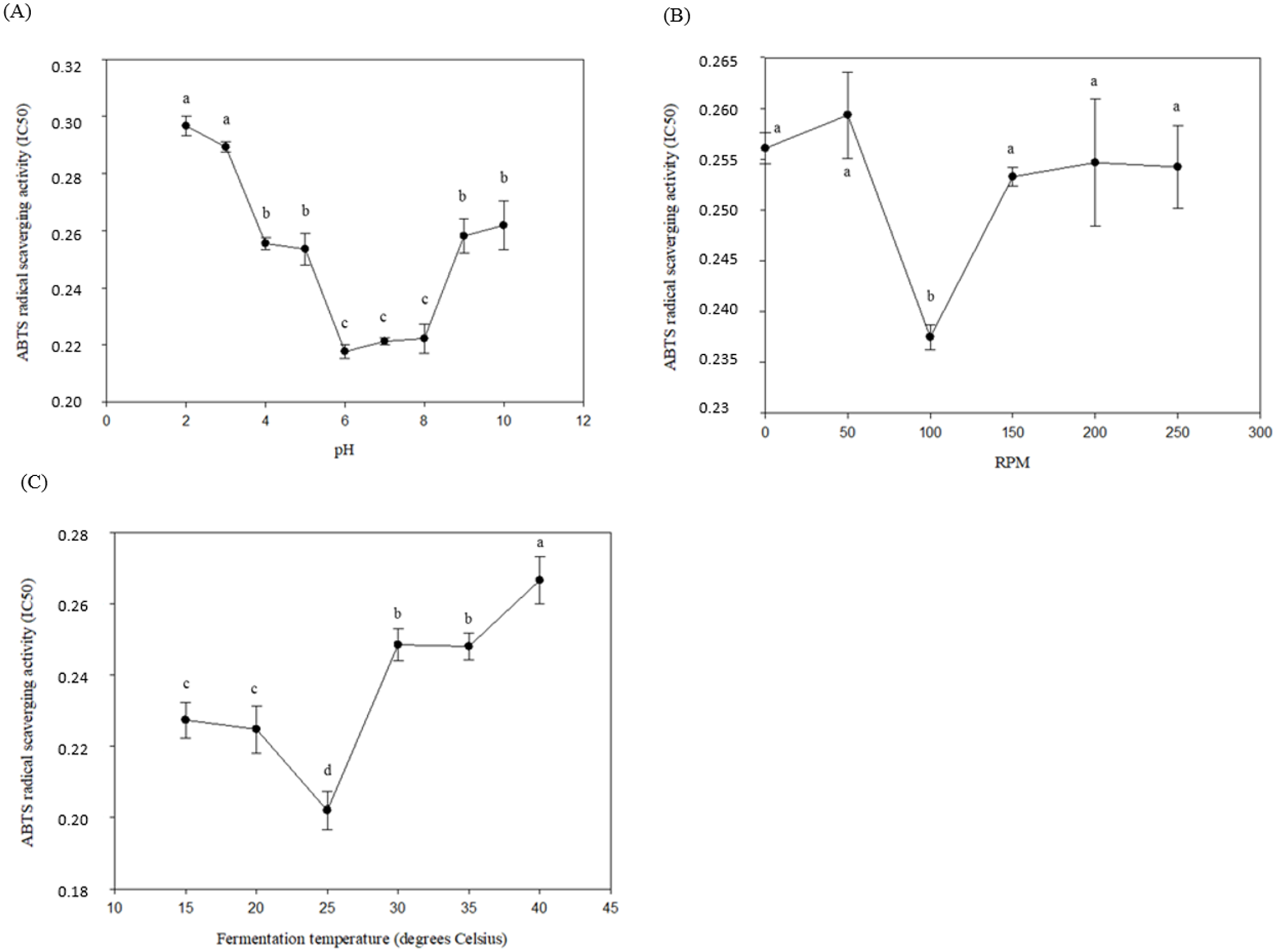

Supplement: S1 Fig — (A) Initial pH-The ABTS radical scavenging activity of Chenopodium formosanum Koidz. fermented with Lactobacillus plantarum BCRC 11697. (B) RPM-The ABTS radical scavenging activity of Chenopodium formosanum Koidz. fermented with Lactobacillus plantarum BCRC 11697. (C) Fermentation temperature-The ABTS radical scavenging activity of Chenopodium formosanum Koidz. fermented with Lactobacillus plantarum BCRC 11697. Statistical differences were calculated by Duncan’s new multiple range test. Values are presented as mean ± SD of three independent experiments with different superscripts (a, b, c, d) are significantly different (p < 0.05). (TIF) [file pone.0249250.s001.tif]

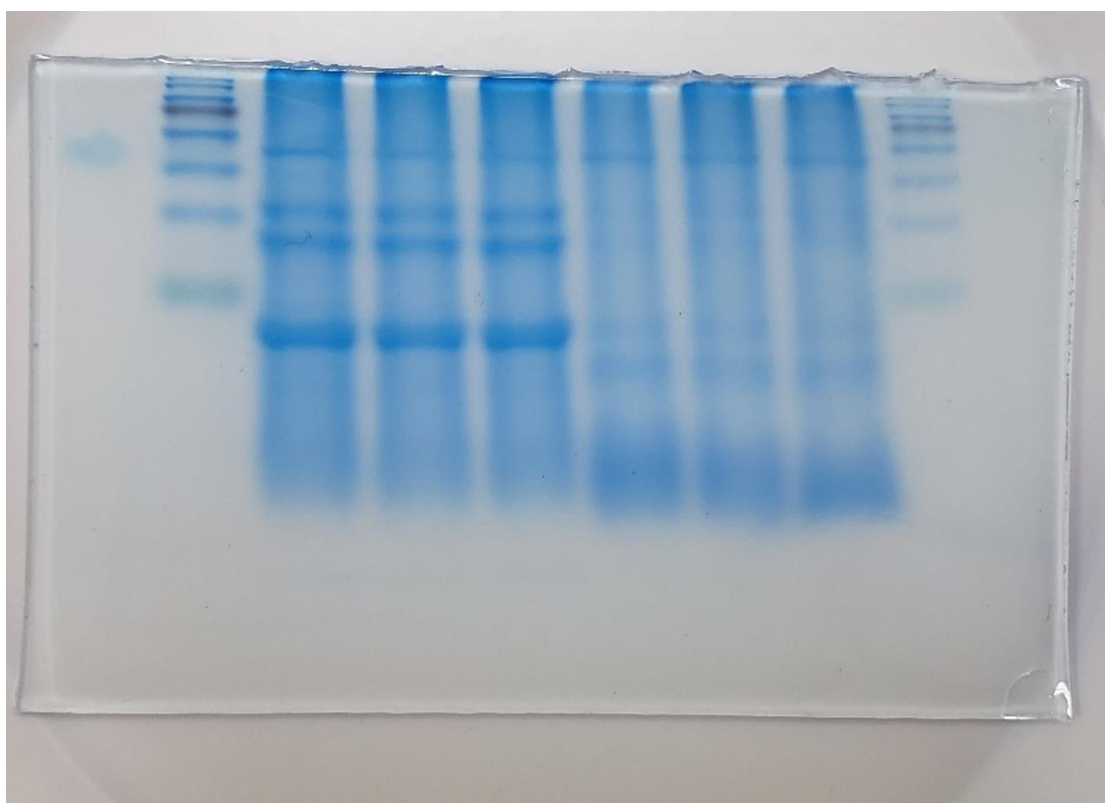

Supplement: S1 Raw image — (PDF) [file pone.0249250.s002.pdf]

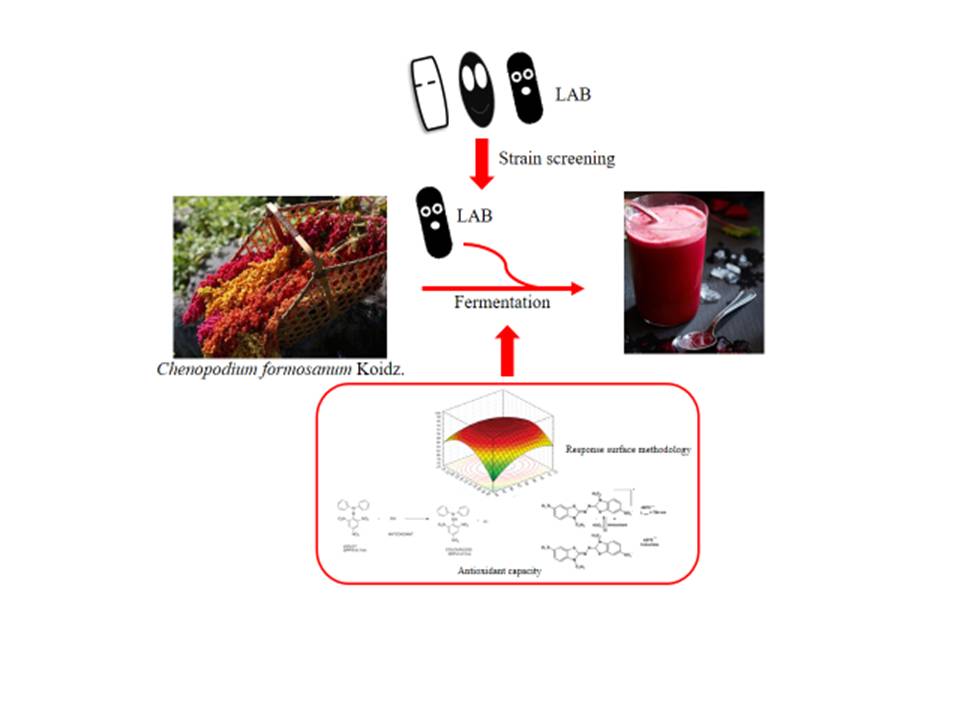

Supplement: S1 Graphical abstract — (JPG) [file pone.0249250.s003.jpg]
