# Supplementary material for: Enhanced antioxidant activity of Chenopodium formosanum Koidz. by lactic acid bacteria: Optimization of fermentation conditions
Source: PLoS One. 2021 May 11;16(5):e0249250. doi: 10.1371/journal.pone.0249250 (PMC8112705; doi:10.1371/journal.pone.0249250)
Supplement: Attachment — Submitted filename: 20210215 Response R2.docx [file pone.0249250.s005.docx]

**Responses to reviewers’ comments**

**Enhanced Antioxidant Activity of *Chenopodium formosanum* Koidz. by Lactic Acid Bacteria: Optimization of Fermentation Conditions**

PONE-D-20-34502R1

**R: "And perhaps explain why they selected ABTS and DPPH assays as the anti-oxidant assays for this study."**

**A: "The reason why we choose ABTS and DPPH assays is because djulis is rich of phenolic compounds."**

**There are numerous antioxidant assays, which differ in their ability to scavenge particular radicals. The authors have to better explain why they have chosen the stated two. It should be discussed in the manuscript too.**

We thank the Academic Editor for the comment, the antioxidant activity is evaluated with the degree of inhibition and blocking of free radicals generated by oxidative stress due to the exposure of the organism to harmful substances such as ethanol, peroxides and chemical agents. The reason why we choose ABTS and DPPH assays is because djulis is rich of phenolic compounds.

Following changes have been made to provide more information about why the assays were used in this study:

Page 12, lines 273-281:

“ABTS and DPPH assays are widely used methods for assessing antioxidant activity in natural herbal products. Both assays are spectrophotometric techniques based on the quenching of stable colored radicals (ABTS or DPPH) and show the radical scavenging ability of antioxidants even when present in complex plant extracts [25]. Both methods are rapid, simple, inexpensive and widely used to measure the ability of compounds to act as free radical scavengers or hydrogen donors, and to evaluate the antioxidant activity of complex extracts. Several studies have also adopted ABTS and DPPH assays to evaluate the quinoa antioxidant activity [17, 26, 27].”

**R: "Results and discussion: In my opinion, this section is not sufficiently elaborated."**

**The authors provided no answer. It is a general remark; however, the Discussion section should be considerably supplemented in several parts where the reviewers' have made their notes.**

We thank the Academic Editor for the comment; however, the comment raised by the reviewer followed by several questions (**i, ii, iii, iv, v, and vi**) which we have been addressed carefully in the response. Following changes have been made to avoid the confusion:

**Page 12, lines 275-289:**

ABTS and DPPH assays are widely used methods for assessing antioxidant activity in natural herbal products. Both assays are spectrophotometric techniques based on the quenching of stable colored radicals (ABTS or DPPH) and show the radical scavenging ability of antioxidants even when present in complex plant extracts [25]. Both methods are rapid, simple, inexpensive and widely used to measure the ability of compounds to act as free radical scavengers or hydrogen donors, and to evaluate the antioxidant activity of complex extracts. Several studies have also adopted ABTS and DPPH assays to evaluate the quinoa antioxidant activity [17, 26, 27].

To determine the optimal djulis fermentation time for *L. plantarum* BCRC 11697, we carried out 54-hour cultivation with samples collected every 6 hours. As shown in Fig 1(A), all LAB strains exhibited similar DPPH scavenging effect, while BCRC11697 showed significantly higher ABTS amongst all the strains (Fig 1(B)); as such, we chose BCRC 11697 for the remainder of the study and used ABTS as the indicator.

**Page 16, lines 336-341:**

The interaction between temperature and agitation speed can be observed from the results (p < 0.05). We hypothesize that is because the heating process can be accelerated by the increased agitation speed, resulting in a favored environment for LAB growth and TPC production. Similar results were reported by Dinarvand et al. [28]. They reported that the interaction between temperature and agitation speed affected the production of invertase from *Aspergillus niger*.

**i) Page 12, line 262 - secrets? or secretes?**

Line 271: We corrected it, thank you. (**secretes**)

**ii) Page 12, line 267-268 - at this stage, why did the authors left out the DPPH-radical scavenging evaluation?**

Following change has been made to clarify the confusion:

**Page 12, lines 285-288:**

Fig 1(A) showed that all LAB strain exhibited similar DPPH scavenging effect while BCRC11697 strain show significantly higher ABTS amongst all strains (Fig 1(B)). Therefore, we choose BCRC11697 for the rest of the study and used ABTS as the indicator.

**iii) Page 12, line 271-274 - this statement is redundant. Similar meaning has been stated in line 259-263. The authors should provide another explanation along with appropriate references.**

We revised it as requested. The sentences have been removed from the text.

**iv) Page 13, line 284 - I wonder why at this stage the authors expressed the results of ABTS scavenging activity in Ic50 but in % at the earlier stage of the study?**

We thank the reviewer for the comment, the data presented in % was in comparison to the control based on the equation: ABTS (%) = [AC - AS] / AC × 100, where AC = ABTS (without samples); AS = Sample + ABTS. We used this method to screen the optimal LAB for djulis fermentation. When the optimal fermentation protocol was evaluated, IC50 was expressed to provide absolute number to the audience.

**v) Page 15, line 336-337 - the sentence 'in the case of quinoa fermentation.....' - please provide reference for this statement.**

**Page 19, lines 349-355:**

**Reference 27** has been added: Xu LA, Guo S, Zhang S. Effects of sloid-state

fermentation with three higher fungion the total phenol contents and antioxidant

properties of diverse cereal grains. FEMS Microbiol Lett. 2018;365:fny163.

**vi) Page 16, line 367-374 - I think that comparison to yogurt fermentation is inappropriate. In my opinion, it is better if the authors compare their results with other studies on cereal or grains fermentation.**

Thanks for your comments. Since we cannot find the similar studies from the plants, the discussion has been removed from the text.

**R: "Conclusions : It is stated in line 384 that the RSM was applied to evaluate the interaction among the three factors. But I could not find the discussion on the interaction of factors anywhere in the manuscript. I can see in Table 3 that the interaction between temperature and agitation speed is significant. The authors should elaborate in the Results and discussion section."**

**A: "Page 14, lines 336-367: The interaction between temperature and agitation speed can be observed from the results (p < 0.05)."**

**I cannot accept the provided statement as the elaboration on the concern raised by the reviewer.**

We thank the Academic Editor for the comment, following discussion has been added:

**Page 16, lines 336-341:**

“The interaction between temperature and agitation speed can be observed from the results (p < 0.05). We hypothesize that is because the heating process can be accelerated by the increased agitation speed, resulting in a favored environment for LAB growth and TPC production. Similar results were reported by Dinarvand et al. [28]. They reported that the interaction between temperature and agitation speed affected the production of invertase from *Aspergillus niger*.”

**A: "We thank the reviewer for the comment, the data presented in % was in comparison to the control based on the equation: DPPH scavenging activity (%) = [1—(A1—A2) / A0] × 100, A0 = DPPH (without samples), A1 = Sample + DPPH, and A2 = Sample (without DPPH). We used this method to screen the optimal LAB for djulis fermentation. When the optimal fermentation protocol was evaluated, IC50 was expressed to provide absolute number to the audience."**

**This reply should stand in the main text. If two out of three reviewers raised the concern on different presentation of scavenging activities, it is expected readers will also do.**

We thank the Academic Editor for the comment, following change has been made:

**Page 7, lines 142-145:**

“We used this method to screen the optimal LAB for djulis fermentation. Once the optimal fermentation protocol was evaluated, the IC50 was calculated to provide an absolute number. IC_50_ of the…”

**The language must be further polished. Some examples:**

**L46-47: "...can significantly increase the phenolic compounds," - phenolic compounds cannot be increased. Its amount can instead.**

**L137 and elsewhere:  "scavenging activity of the djulis" - No article is used when a non-count noun is generic or nonspecific.**

**L244: "T test" it is rather "t- test"**

**L363: no contractions such as "it's" are allowed.**

We thank the Academic Editor for the comment. The revision has been sent for the English proofreading and the certificate has been attached as supplemental document.

**Table titles and figure captions: please do not write djulis capitalized.**

We have fixed it as requested by the Academic Editor.

**Figures 3 and 4 should be submitted in the .tiff format. Figure S1 is not a raw image since has been edited.**

Figure 3 and 4 have been submitted in the .tiff format. A raw image of Fig. S1 has been uploaded as requested.

**Significance letters in three histograms representing Figure 1 might be questionable. Please provide statistical working sheet with the raw data as Supporting information.**

We thank the Academic Editor for pointing out the issue. The data set of Fig. 1 has been analyzed carefully using one-way ANOVA. Following change has been made to avoid the confusion. A statistical working sheet with the raw data has also been uploaded as supporting information.

Page 11, line 245:

“…was used for **one-way ANOVA** and Duncan’s new multiple range…”

**Response to the reviewers:**

**-------------------------------------------------------------------------------------------------------**

**Authors should pay more attention to the aims of the study (Introduction section) and how they present results in the Results section. Calculation methods of antioxidant assays may be questionable. Some statistical significance might be presented in an inappropriate manner, as noted in the Reviewers' reports. Labels in Fig 1A and Fig 1B are not visible. Can they stay in an oblique way, as in Fig 1C? Figure 3 should be prepared according to Submission Guidelines specified for figures:** <https://journals.plos.org/plosone/s/figures>.

We thank editor for the comments. Lables of Figure 1A and 1B have been fixed as requested and Figure 3 has been prepared to meet the requirment of **Submission Guidelines.** All the comments mentioned above have been addressed carefully in the response.

**Language needs considerable improvement.**

We thank editor for the suggestion. Our manuscript has been proofread carefully by a native speaker (Iris YS Wu) from Molecular Environmental Biology, University of California, Berkeley (Berkeley, CA, USA)**.** Dr. Shu-Han Yu who received her PhD degree from Johns Hopkins University, School of Medicine and Prof. Kuan-Chen Cheng who received his PhD degree from the Pennsylvania State University also helped to finalize the revision. We also sent our manuscript for English proofreading service.

**Journal Requirements:**

**When submitting your revision, we need you to address these additional requirements.**

**1. Please ensure that your manuscript meets PLOS ONE's style requirements, including those for file naming. The PLOS ONE style templates can be found at**

**https://journals.plos.org/plosone/s/file?id=wjVg/PLOSOne_formatting_sample_main_body.pdf and**

**https://journals.plos.org/plosone/s/file?id=ba62/PLOSOne_formatting_sample_title_authors_affiliations.pdf**

Thanks for suggestion, we already revised to meet PLOS ONE's style requirements, including heading, sub-heading, style of references and so on.

**2. Please include your tables as part of your main manuscript and remove the individual files. Please note that supplementary tables should be uploaded as separate "supporting information" files.**

 Thanks for reminding, the supporting information have been separated.

**3. Thank you for stating the following in the Acknowledgments Section of your manuscript:**

**"This project was funded by the Ministry of Science and Technology, Taiwan (MOST
 109-2628-E-002-007-MY3 and MOST 107-2320-B-255-001-MY3). The funding
grant of BMRPD42 for this study was also provided in part by research grants from the Chang Gung Memorial Hospital, Chiayi, Taiwan."**

**We note that you have provided funding information that is not currently declared in your Funding Statement. However, funding information should not appear in the Acknowledgments section or other areas of your manuscript. We will only publish funding information present in the Funding Statement section of the online submission form.**

**Please remove any funding-related text from the manuscript and let us know how you would like to update your Funding Statement. Currently, your Funding Statement reads as follows:**

**"This project was funded by the Ministry of Science and Technology, Taiwan (MOST 109-2628-E-002-007-MY3 and MOST 107-2320-B-255-001-MY3). HC Kuo received grant of MOST 107-2320-B-255-001-MY3. KC Cheng received grant of MOST 109-2628-E-002-007-MY3. URL: https://www.most.gov.tw/?l=en . The funders had no role in study design, data collection and analysis, decision to publish, or preparation of the manuscript."**

**Please include your amended statements within your cover letter; we will change the online submission form on your behalf.**

The funding sources have been removed from the **Acknowledgments section.**

Following changes has been made:

**The authors greatly acknowledge the article proofreading by Iris YS Wu who is a native speaker from Molecular Environmental Biology, University of California, Berkeley (Berkeley, CA, USA).**

**4. PLOS ONE now requires that authors provide the original uncropped and unadjusted images underlying all blot or gel results reported in a submission’s figures or Supporting Information files. This policy and the journal’s other requirements for blot/gel reporting and figure preparation are described in detail at https://journals.plos.org/plosone/s/figures#loc-blot-and-gel-reporting-requirements and https://journals.plos.org/plosone/s/figures#loc-preparing-figures-from-image-files. When you submit your revised manuscript, please ensure that your figures adhere fully to these guidelines and provide the original underlying images for all blot or gel data reported in your submission. See the following link for instructions on providing the original image data: https://journals.plos.org/plosone/s/figures#loc-original-images-for-blots-and-gels.**

**In your cover letter, please note whether your blot/gel image data are in Supporting Information or posted at a public data repository, provide the repository URL if relevant, and provide specific details as to which raw blot/gel images, if any, are not available. Email us at plosone@plos.org if you have any questions.**

We have provided our original, uncropped gel image along with our revision. It’s in

**Supporting Information.**

**5. Please include captions for your Supporting Information files at the end of your manuscript, and update any in-text citations to match accordingly. Please see our Supporting Information guidelines for more information:** [**http://journals.plos.org/plosone/s/supporting-information**](about:blank)**.**

The captions of supplemental figure 1 and blot image have been provided at the end of the manuscript.

**Comments to the Author**

**Reviewer #1: Overall, the manuscript provides a good information of the subject matter. The experimental designs and analyses are standard and appropriate for the study. However, there are some concerns that need to be addressed by the authors.**

**1. The authors may wish to send the manuscript for a professional proof-read. There are some grammatical errors and inconsistency of sentences. Many of the scientific names of organisms throughout the manuscript are not in italic.**
Our manuscript has been proofread carefully by a native speaker (Iris YS Wu) from Molecular Environmental Biology, University of California, Berkeley (Berkeley, CA, USA)**.** Dr. Shu-Han Yu who received her PhD degree from Johns Hopkins University, School of Medicine and Prof. Kuan-Chen Cheng who received his PhD degree from the Pennsylvania State University also help to finalize the revision.

**2. Introduction:
i) The sentence 'Lactic acid bacteria (LAB) are responsible for a group of famous strains of probiotics' (page 4, line 62-63) is somewhat misleading.**

Thank you, we had modified it as: **Lactic acid bacteria (LAB) are famous strains of probiotics. (page 4, line 67-68)**

**ii) The motivation/cause for this study is not clearly explained. The authors may wish to further explain the reason why they wanted to enhance the antioxidant activity and also why they selected LAB in the fermentation process. And perhaps explain why they selected ABTS and DPPH assays as the anti-oxidant assays for this study.**
Thanks for the comments.

1. The motivation of this study is trying to elucidate the best Djulis fermentation condition among the different strains of LAB.
2. Djulis have been utilized by Taiwan aboriginals for different purposes, and it could be made to LAB beverages in the future from our study. Our results can improve economic value of Djulis industry.
3. The antioxidant activity is evaluated with the degree of inhibition and blocking of free radicals generated by oxidative stress due to the exposure of the organism to harmful substances such as ethanol, peroxides and chemical agents. The reason why we choose ABTS and DPPH assays is because djulis is rich of phenolic compounds.

Following changes have been made to clarify this concern:

Page 3, lines 51-52:

**The results indicated that djulis fermented by *L*. *plantarum* can be a powerful source of natural antioxidants for the prevention of free radical-initiated diseases**.

**Page 5, line 87:**

“…enhance antioxidant activity **for the development of health-promoting beverages.**”

**3. Materials and methods:**

**i) Page 7, line 143 - ABTS full name is repetitive.**

We have removed the redundant term.

**ii) Page 9, line 193 - stated here that the authors used SAS software. But on page 13, line 300 - the authors stated that they used Minitab. Which statistical software did they actually use? Why did the authors used two different statistical software?**
We are sorry for the confusion. Following change has been made:

Page 9, line 199: A **Minitab** software was used to predict the…

Page 11, lines 243-245: Minitab Statistical Software (Minitab Inc., University City, Pennsylvania, USA) was used **for T test and Duncan’s new multiple range tests and RSM evaluation and analysis**.

**4. Results and discussion: In my opinion, this section is not sufficiently elaborated.
i) Page 12, line 262 - secrets? or secretes?**

Line 271: We corrected it, thank you. (**secretes**)

**ii) Page 12, line 267-268 - at this stage, why did the authors left out the DPPH-radical scavenging evaluation?**

Following change has been made to clarify the confusion:

Page 12, lines 285-288:

Fig 1(A) showed that all LAB strain exhibited similar DPPH scavenging effect while BCRC11697 strain show significantly higher ABTS amongst all strains (Fig 1(B)). Therefore, we choose BCRC11697 for the rest of the study and used ABTS as the indicator.

**iii) Page 12, line 271-274 - this statement is redundant. Similar meaning has been stated in line 259-263. The authors should provide another explanation along with appropriate references.**

We revised it as requested. The sentences have been removed from the text.

**iv) Page 13, line 284 - I wonder why at this stage the authors expressed the results of ABTS scavenging activity in Ic50 but in % at the earlier stage of the study?**

We thank the reviewer for the comment, the data presented in % was in comparison to the control based on the equation: ABTS (%) = [AC—AS] / AC × 100, where AC = ABTS (without samples); AS = Sample + ABTS. We used this method to screen the optimal LAB for djulis fermentation. When the optimal fermentation protocol was evaluated, IC50 was expressed to provide absolute number to the audience.

**v) Page 15, line 336-337 - the sentence 'in the case of quinoa fermentation.....' - please provide reference for this statement.**

Page 19, lines 349-355:

**Reference 27** has been added: Xu LA, Guo S, Zhang S. Effects of sloid-state

fermentation with three higher fungion the total phenol contents and antioxidant

properties of diverse cereal grains. FEMS Microbiol Lett. 2018;365:fny163.

**vi) Page 16, line 367-374 - I think that comparison to yogurt fermentation is inappropriate. In my opinion, it is better if the authors compare their results with other studies on cereal or grains fermentation.**

Thanks for your comments. Since we cannot find the similar studies from the plants, the discussion has been removed from the text.

**5. Conclusions : It is stated in line 384 that the RSM was applied to evaluate the interaction among the three factors. But I could not find the discussion on the interaction of factors anywhere in the manuscript. I can see in Table 3 that the interaction between temperature and agitation speed is significant. The authors should elaborate in the Results and discussion section.**
Following change has been made as requested:

Page 14, lines 323-328:

“The interaction between temperature and agitation speed can be observed from the results (p < 0.05). We hypothesize that is because the heating process can be accelerated by the increased agitation speed, resulting in a favored environment for LAB growth and TPC production. Similar results were reported by Dinarvand et al. [28]. They reported that the interaction between temperature and agitation speed affected the production of invertase from *Aspergillus niger*.”

**6. Table 4: Are the superscript letters indicating significance of the data are stated correctly? Please check.**

Thanks for the comment, we have modified it accordingly.

**Reviewer #2: Reviewer
The research article entitled “Enhanced Antioxidant Activity of Chenopodium formosanum Koidz. by Lactic Acid Bacteria: Optimization of Fermentation Conditions” submitted in your esteemed journal is a good work where the authors have tried to improve the antioxidant activity by lactic acid bacterial and also optimized the fermentation conditions.**

**Minor Comments: -
What is the method of preparation of bacterial cell suspensions used for fermentation? Also describe about the starter culture for fermentation in material and method section.**

Thanks for this comment, we revised it in the material section.

Page 6, lines 115-116 and lines 123-124:

**“Viable cells were grown in MRS medium at 37°C for 20 hours as inoculum** and sub-cultured twice a month **[16]**.”

“…sample was inoculated with **1% of LABs (~7 log CFU ml ^-1^)** **as seed culture** mentioned above and fermented at 37°C for 48 hours.”

**In DPPH section what is 1 in the calculation of scavenging activity (%)?
Author cite only one reference for both DPPH and ABTS assay but author used two different calculation method for both DPPH as well as ABTS. Be specific about the citations as well as the calculation.**

We thank the reviewer for the comment, the data presented in % was in comparison to the control based on the equation: DPPH scavenging activity (%) = [1—(A1—A2) / A0] × 100, A0 = DPPH (without samples), A1 = Sample + DPPH, and A2 = Sample (without DPPH). We used this method to screen the optimal LAB for djulis fermentation. When the optimal fermentation protocol was evaluated, IC50 was expressed to provide absolute number to the audience.

**In section 2.5 author mentioned Wu et al (2020). What is the number of this citation in final list of references?**

The Wu et al here refers to Ref 13, which has been fixed to avoid confusion.

**In section 2.7 line space is different as compared to the other text. Kindly follow the uniformity in whole MS.**

Thank you for the comment. We have revised our manuscript carefully to follow the PLOS One regulation.

**The figures are not clearly visible.**

We thank reviewer for the comments. Lables of Figure 1A and 1B have been fixed as requested.

**At line no 310 author highlights the bracket with red colour. Correct it accordingly.**

Thanks, we corrected it.

**From section 3.3 it showed that fermentation decrease the carbohydrate, protein and fat content. Kindly explain? What is the reason behind this?**

We thank the reviewer for the comment. Following change has been made:

Page 15, lines 362-366:

**LAB utilized the carbohydrate, protein and fat of djulis since it’s the only nutrient within the medium. Some of them could be hydrolyzed into small molecules such as peptides, oligosaccharides and short chain fatty acids due to the presence of related enzymes.**

**In table 4 correct the word TPC instead of TCP.**

Thanks for comment, we corrected it.

**Correct the MS with a uniform pattern according to the journal guidelines and also formatting it accordingly.**

Thanks for kindly reminding, we already meet PLOS ONE's style requirements, including heading, sub-heading, style of references and so on.

**“After reviewing the manuscript, in my opinion that once the corrections will amended by the author than it will be fit and accepted for publication in your esteemed journal”.**

**Reviewer #3: The paper describes the utilization of RSM in the optimization of fermentation parameters in order to maximize antioxidant activity. The work is scientifically interesting and sound.
It is good that the authors point out that the increase in antioxidant capacity may be partly related to the enhanced extractability of phenolic compounds. It needs to be remembered that human gut is able to effiicently "extract" and utilize also phenolic compounds that - due to non-extractability in sample preparation - may not be observed when total phenolics/antixoidant capacity is measured.**

Thanks for your opinion; this is one of our future challenges based on the results of this study.

**English needs to be slightly improved.**

Our manuscript has been proofread carefully by a native speaker (Iris YS Wu) from Molecular Environmental Biology, University of California, Berkeley (Berkeley, CA, USA)**.** Dr. Shu-Han Yu who received her PhD degree from Johns Hopkins University, School of Medicine and Prof. Kuan-Chen Cheng who received his PhD degree from the Pennsylvania State University also help to finalize the revision.

**Some clarification is needed in the following:
1) What is the difference between "Fermented" and "Fermented-RSM" in Table 4?**

Thanks for the comment, following footnote has been added in Table 4: (Page 18)

**Unfermented: unfermented Djulis; Fermented: Djulis fermented by *L. plantarum* BCRC11697; Fermented-RSM: Djulis fermented by *L. plantarum* BCRC11697 after RSM optimization.**

**2) What is "quinoa" mentioned in Table 5? Reference to literature source may be needed. Does "Djulis" denote the nonfermented samples?**

Sorry for the confusion. We analyzed those samples by ourselves. Quinoa and djulis denote the nonfermented samples. Following changes have been made:

Page 11, line 230:

**“Quinoa, djulis and fermented djulis** samples were analyzed…”
